# Inhibition of endosomal trafficking by brefeldin A interferes with long‐distance interaction between chloroplasts and plasma membrane transporters

**DOI:** 10.1111/ppl.13058

**Published:** 2019-12-26

**Authors:** Alexander A. Bulychev, Ilse Foissner

**Affiliations:** ^1^ Department of Biophysics, Faculty of Biology Moscow State University Moscow Russia; ^2^ Department of Biosciences University of Salzburg Salzburg Austria

## Abstract

The huge internodal cells of the characean green algae are a convenient model to study long‐range interactions between organelles via cytoplasmic streaming. It has been shown previously that photometabolites and reactive oxygen species released by illuminated chloroplasts are transmitted to remote shaded regions where they interfere with photosynthetic electron transport and the differential activity of plasma membrane transporters, and recent findings indicated the involvement of organelle trafficking pathways. In the present study, we applied pulse amplitude‐modulated microscopy and pH‐sensitive electrodes to study the effect of brefeldin A (BFA), an inhibitor of vesicle trafficking, on long‐distance interactions in *Chara australis* internodal cells. These data were compared with BFA‐induced changes in organelle number, size and distribution using fluorescent dyes and confocal laser scanning microscopy. We found that BFA completely and immediately inhibited endocytosis in internodal cells and induced the aggregation of organelles into BFA compartments within 30–120 min of treatment. The comparison with the physiological data suggests that the early response, the arrest of endocytosis, is related to the attenuation of differences in surface pH, whereas the longer lasting formation of BFA compartments is probably responsible for the acceleration of the cyclosis‐mediated interaction between chloroplasts. These data indicate that intracellular turnover of membrane material might be important for the circulation of electric currents between functionally distinct regions in illuminated characean internodes and that translational movement of metabolites is delayed by transient binding of the transported substances to organelles.

**Abbreviations**
AFW: artificial fresh water
AOI: analyzed area of inspection
BFA: brefeldin A
BGL: background light
Chl: chlorophyll
DMSO: dimethyl sulfoxide
ER: endoplasmic reticulum
F′: actual Chl fluorescence in a cell exposed to actinic light
Fm′: maximal Chl fluorescence yields in cells exposed to actinic light
FM 1‐43FX: N‐(3‐triethylammoniumpropyl)‐4‐(4‐(dibutylamino)styryl)pyridinium dibromide
IAA: indole‐3‐acetic acid
LED: light‐emitting diode
LL: local light
MVB: multivesicular body
PAM: pulse amplitude‐modulated microscopy
PFD: photon flux density
pH_o_: pH at the cell surface
PQ: plastoquinone
PS: photosystem
TGN: *trans*‐Golgi network

## Introduction

The characean internodal cells generate alternating patterns of low and high pH along their surface (pH_o_; Fig. S1A; reviewed by Beilby and Bisson 2012 and Beilby 2016). The pH banding pattern correlates with the photosynthetic activity of the anchored chloroplasts being high at the acidic regions because of enhanced availability of carbon dioxide (Fig. S1B; Plieth et al. 1994, Bulychev and Vredenberg 2003). The acidification of the external medium is because of the activity of plasma membrane located H^+^ ATPases that pump cytoplasmic protons outward. To maintain pH homeostasis of the cytoplasm, outward directed proton current is balanced by H^+^ influx or OH^−^ efflux (high pH channels; Bisson and Walker 1980). Previous studies suggest that the high pH channels are activated by metabolites released from photosynthetically active chloroplasts (Eremin et al. 2007, Bulychev and Komarova 2014).

There is evidence that the intracellular turnover of membrane material might be important for the circulation of electric currents between functionally distinct regions in illuminated characean internodes and for long‐distance signaling within the cell. For example, an inhibitor of cytoplasmic vesicle trafficking, wortmannin suppressed the pH banding in *Chara* and eliminated a minor component in the cyclosis‐mediated changes of chlorophyll (Chl) fluorescence (Bulychev and Foissner 2017). The action of other inhibitors of vesicular transport, e.g. brefeldin A (BFA), on proton flows and Chl fluorescence changes is of interest. The alteration in the abundance or turnover rates of cytoplasmic vesicles may restrict the intracellular mobility of laterally transported solutes through binding and the release of these solutes to membrane vesicles. Boot et al. (2012) observed the polar movement of indole‐3‐acetic acid (IAA) along *Chara* internodes at velocities comparable to or lower than the rate of cytoplasmic streaming. However, IAA transport was insensitive to inhibition of cyclosis, unlike metabolite signaling detected with pulse amplitude‐modulated microscopy (PAM) fluorometry (Bulychev and Foissner 2017).

The interactions between the endosomal trafficking, lateral transmission of metabolites and the formation of banding pattern are hardly investigated to date. Recent studies revealed similarities and distinctions in the effects of BFA and wortmannin on structure and function of plant cells (Bulychev et al. 2018). In this study, we examined the influence of BFA on light‐dependent proton flows across the plasma membrane and on the cyclosis‐mediated regulation of photosystem (PS) II activity in *Chara* internodal cells. Long‐distance signaling was investigated by the application of local illumination and measuring Chl fluorescence at non‐irradiated downstream areas (Fig. S1C). Such experiments have previously revealed that chloroplast electron transport can be modified by reducing equivalents released from illuminated regions and transported to non‐illuminated chloroplasts via the streaming endoplasm (Fig. S1C; Bulychev et al. 2013, Bulychev and Komarova 2015, 2017).

BFA is a macrocyclic lactone produced by a range of fungi belonging to different genera (Wang et al. 2002) and responsible for inducing leaf spot disease in susceptible plants (Tietjen et al. 1985). The interest in BFA initially focused on its antiviral and antitumor activities (Tamura et al. 1968) which were, however, insufficient for clinical application. Later, it became clear that BFA is a useful tool for investigating vesicle trafficking in animal (Takatsuki and Tamura 1985) and plant cells (Satiat‐Jeunemaitre and Hawes 1992a, b). Since then, BFA has become widely used in the study of membrane pathways toward and away from the plasma membrane. The target of BFA are guanine‐nucleotide exchange factors which activate ADP‐ribosylation factor proteins, small GTPases, involved in the recruitment of membrane coats required for cargo sorting and for the release of vesicles (see Singh and Jürgens 2018 for review). A common feature of BFA treatment is the formation of BFA compartments or BFA bodies (Satiat‐Jeunemaitre and Hawes 1992b). They usually consist of agglomerations of Golgi bodies and/or *trans*‐Golgi network (TGN), or of their remnants, and may include aggregates of endoplasmic reticulum (ER) cisternae (Staehelin and Driouich 1997, Nebenführ et al. 2002, Ritzenthaler et al. 2002, Robinson et al. 2008). In most plant cells investigated so far, BFA predominantly inhibits the release of Golgi and TGN vesicles and their fusion with the plasma membrane (exocytosis; Naramoto et al. 2010). In *Nicotiana* BY2 cells, BFA has been reported to arrest the release of endosomes from the plasma membrane (endocytosis; Jelinkova et al. 2015).

In the present study, we investigated the effect of BFA on the multicellular green alga *Chara*. We found that BFA completely and immediately inhibited endocytosis in internodal cells and induced the formation of BFA compartments within 30–120 min of treatment. The comparison with the physiological data suggests that the early response, the arrest of endocytosis, is related to changes in pH banding, whereas the longer lasting agglomeration and fusion of organelles is probably responsible for the acceleration of the cyclosis‐mediated interaction between chloroplasts, owing to reduced binding of metabolites to membrane surfaces.

## Materials and methods

### Algal material, culture conditions and inhibitor treatment

Thalli of *Chara australis* R.Br. were grown as described previously (Foissner et al. 2016). Internodal cells were isolated from the main axis with a small pair of scissors and left in artificial fresh water (AFW; 10^−3^ M NaCl, 10^−4^ M KCl and 10^−4^ M CaCl_2_) until use.

BFA (BFA; Sigma Aldrich) was dissolved in dimethyl sulfoxide (DMSO) at a concentration of 70 mM. The stock solution was diluted with AFW and the control solutions contained the appropriate amount of DMSO.

### pH electrode measurements

Alkaline and acid bands were identified with tip‐sensitive antimony pH microelectrodes as described by Bulychev et al. (2001).

### Chl fluorescence measurements

Chl fluorescence was measured on microscopic cell regions (∼100 µm in diameter) with a Microscopy PAM fluorometer (Walz) combined with an Zeiss Axiovert 25 CFL inverted microscope. Weak measuring light from the blue light‐emitting diode (LED) of the PAM directed through the microscope optical path excited minimal fluorescence (F_o_) measured in dark‐adapted cells and fluorescence (F′, actual Chl fluorescence in a cell exposed to actinic light) observed under dim background illumination. Maximal Chl fluorescence yields in dark‐adapted cells (Fm) and in cells exposed to actinic light (Fm′) were induced by saturating light pulses. The signal from the photomultiplier was processed with WinControl‐3 software (Walz). It was also digitized with a PCI‐6024E AD‐converter (National Instruments) and displayed on a computer. Data points were sampled at intervals of ∼51 ms.

The whole cell was continuously exposed to dim background light (BGL). This light was directed from the upper illuminator of the Axiovert 25 CFL microscope and passed through a blue glass filter (SZS‐22, λ < 580 nm; Lytkarino Optical Glass Factory) and a neutral density glass filter. The photon flux density (PFD) was 12.5 µmol m^−2^ s^−1^ in most cases. Low‐intensity BGL keeps light‐dependent enzymes in active condition but does not induce energy‐dependent non‐photochemical quenching. To promote the formation of pH banding pattern, the cells were first exposed to BGL of higher intensity (35 µmol m^−2^ s^−1^) and then the photon flux was attenuated to values sufficient for the maintenance of pH bands.

Cyclosis‐mediated transmission of photometabolites was assessed by measuring Chl fluorescence F′ after applying a 30‐s pulse of local light (LL) at a distance of 1–1.5 mm on the upstream side from the analyzed area of inspection (AOI; Fig. S1). The delivery of photometabolites to the shaded green plastids induced the delayed rise of F′ in AOI. The LL pulse was applied through a quartz optic fiber (400 µm diameter) from a computer‐controlled LED source of white light. The PFD at the output was ∼500 µmol m^−2^ s^−1^. The tip of the fiber optic light guide was first placed near the cell and then displaced with a micrometric screw to a required distance d (usually 1.5 mm) upstream of the cytoplasmic flow with respect to AOI.

### In vivo staining of organelles and confocal laser scanning microscopy

For in vivo staining of the plasma membrane and endocytic organelles, internodal cells were pulse labeled for 2–4 min with 10 µM green fluorescent FM 1‐43FX (N‐(3‐triethylammoniumpropyl)‐4‐(4‐(dibutylamino)styryl)pyridinium dibromide) (Thermo Fisher) dissolved in AFW. The Chl autofluorescence of the cortical chloroplasts impedes the visualization of fluorescent organelles in the streaming endoplasm of *Chara* internodal cells. Therefore, chloroplasts were removed locally by irradiation with intense (chloroplast damaging) blue light of a mercury or halide lamp guided through the filter cube of a fluorescence microscope (Kamitsubo 1972). Cells with chloroplast‐free ‘windows’ were allowed to recover cytoplasmic streaming at least 1 day prior to experiments.

A Leica TCS SP5 confocal system coupled to a DMI 6000B inverted microscope was used to visualize FM 1‐43‐fluorescent organelles. Videos (60 frames) of the streaming endoplasm were taken with maximum speed (1000 Hz) using a 63× water immersion objective (numerical aperture 1.2) and an HyD sensor. Relative number and areas of endosomes were calculated from single frames using imagej (https://imagej.nih.gov/). Diagrams were produced in microsoft excel (https://products.office.com).

## Results

### Microfluidic control of pH_o_ and Chl fluorescence in the alkaline and acid bands

**Figure 1:**
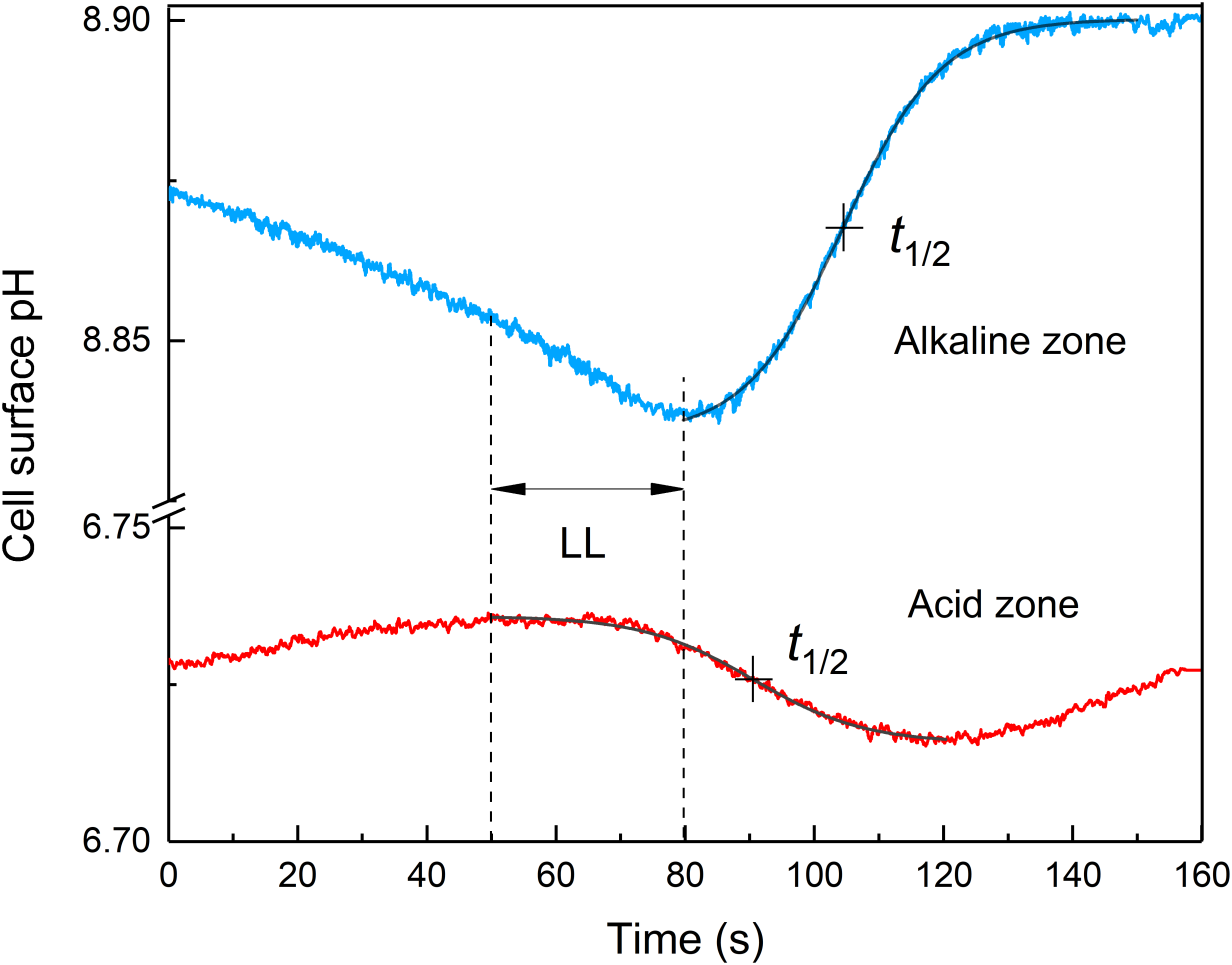
Transient pH changes in alkaline and acid zones on the surface of *Chara* internodal cell in response to local illumination of a cell region located 1.5 mm upstream from the analyzed area. Note that the application of an LL pulse promoted the increase and decrease in pH_o_ in the alkaline and acidic zones, respectively. The frontal parts of pH transients were approximated with Boltzmann sigmoid curves (smooth black lines superimposed on the experimental curves). Dashed vertical lines indicate the time of LL application (from 50 to 80 s). Positions of t_1/2_ for pH changes are marked with crosses.

Under dim background irradiance in the absence of inhibitors, the pH in alkaline zones on the surface of *Chara* internodes was slightly lower than at high irradiance but it increased upon local illumination (LL) of a nearby cell region on the upstream side of the analyzed area (Fig. S1C). As can be seen in Fig. 1, the application of LL pulse to a remote area (at a fixed distance d from the point of measurement) induced pH changes of opposite signs in the alkaline and acid regions: the pH increased after a lag period in the alkaline zone and dropped transiently in the acid region. We characterized these pH changes by the time t_1/2_ counted from the onset of LL pulse to the moment when pH shifted by half between the minimum and maximum levels (Fig. 1). The time t_1/2_ was routinely determined by approximating the pH transients with the Boltzmann sigmoid curve. Subtraction of the baseline drift had no appreciable influence on the results of t_1/2_ determination.

**Figure 2:**
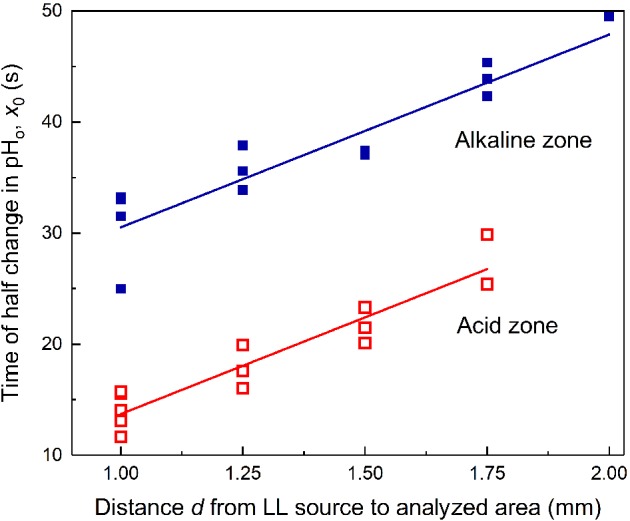
Time of attaining the half‐maximum pH_o_ change in the alkaline and acid zones counted from the onset of LL pulse as a function of distance d between the optic fiber and the point of pH_o_ measurement.

The plots of time t_1/2_ for LL‐induced pH transients as a function of the distance d from the illuminated spot contain information on the mechanisms by which the regulatory metabolites travel along the cell. Specifically, a linear shape of t_1/2_(d) graphs points to the cyclosis‐mediated movement, whereas a quadratic dependence is typical for diffusion. Fig. 2 shows that the plots of t_1/2_ as a function of separation distance d were linear for the LL‐induced pH changes both in the alkaline and acid zones. Both plots had equal slopes (17.4 ± 2.0 s mm^−1^) but were shifted along the ordinate by ∼15 s.

The inverse of the slope for these curves (57.5 µm s^−1^) corresponds to the rate at which the regulatory signals traveled along the cell. This signal transmission rate is comparable to but somewhat lower than the values of 70–90 µm s^−1^ typical of cyclosis velocity. The shift of these two curves along the y‐axis might indicate the existence of two different mediators that are released from illuminated chloroplasts without a discernible delay (activator of acid zone) and after a 15‐s lag period (activator of the alkaline zone).

**Figure 3:**
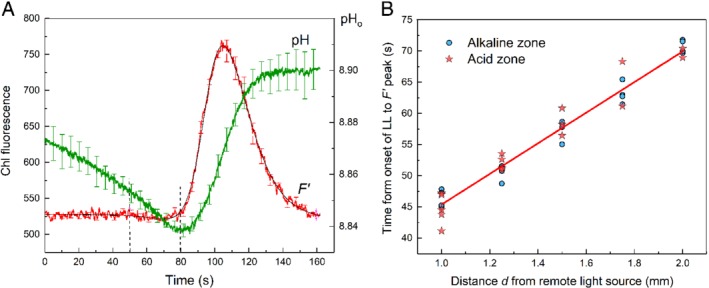
Changes in Chl fluorescence and pH_o_ induced by a 30‐s illumination of a remote (d = 1.5 mm) cell region (A) and the plots of the F′ peak position (counted from the onset of LL pulse) against the distance between the LL source and the analyzed area underlying the acid and alkaline zones (B). The smooth thin line in (A) is the approximation of experimental data with two Boltzmann functions. Average traces of F′ and pH_o_ (±se) were obtained for n = 4 in an experiment with a representative cell. Dashed vertical lines indicate the time of LL application (from 50 to 80 s).

The local illumination also affected Chl fluorescence emission in chloroplasts located downstream in the fluid flow (Fig. 3A). Illuminated chloroplasts are known to release reducing equivalents (NADPH and triose phosphates) into the cytoplasm (Taniguchi and Miyake 2012), which travel with the streaming fluid and are possibly imported by recipient chloroplasts in shaded cell areas (Selinski and Scheibe 2019). After the entry of reducing substances into the stroma, they promote the reduction of intersystem electron transport carriers, which is evident in the transient rise of Chl fluorescence. As can be seen in Fig. 3A, the peak of F′ response fell into the time range where the ascending front of pH_o_ occurred. The time to F′ peak (counted from the onset of LL) increased linearly with the distance d between the LL source and the detection area. The plots for F′ peak position, t_p_ as a function of distance d in cell regions under the acid and alkaline bands, were essentially similar (Fig. 3B).

In cell regions with extreme alkaline values (external pH ∼ 10), the fluorescence responses to distant LL were strongly suppressed and unsuitable for analysis. Because of this, we performed measurements at cell regions with pH_o_ 8.5–8.8, at which the F′ fluorescence response to LL is well pronounced. The plots for the alkaline and acid zones had similar slopes and showed no significant shift along the y‐axis. The intercept value at d = 0 for the fluorescence plot (20.85 ± 1.5 s) was slightly larger than for the pH plot in the alkaline zone (13.2 ± 3.0 s). The intercept value represents the total time during which the regulatory metabolite is generated and processed except for the time of its transportation with the fluid flow. A shorter time for microfluidic chloroplast–plasmalemma interactions compared to interchloroplast interactions is reasonable because the interchloroplast communications involve an additional stage of translocation across the envelope membrane of recipient chloroplasts.

### Influence of BFA on pH banding and cyclosis‐mediated regulation of H^+^ (OH^−^) channels

**Figure 4:**
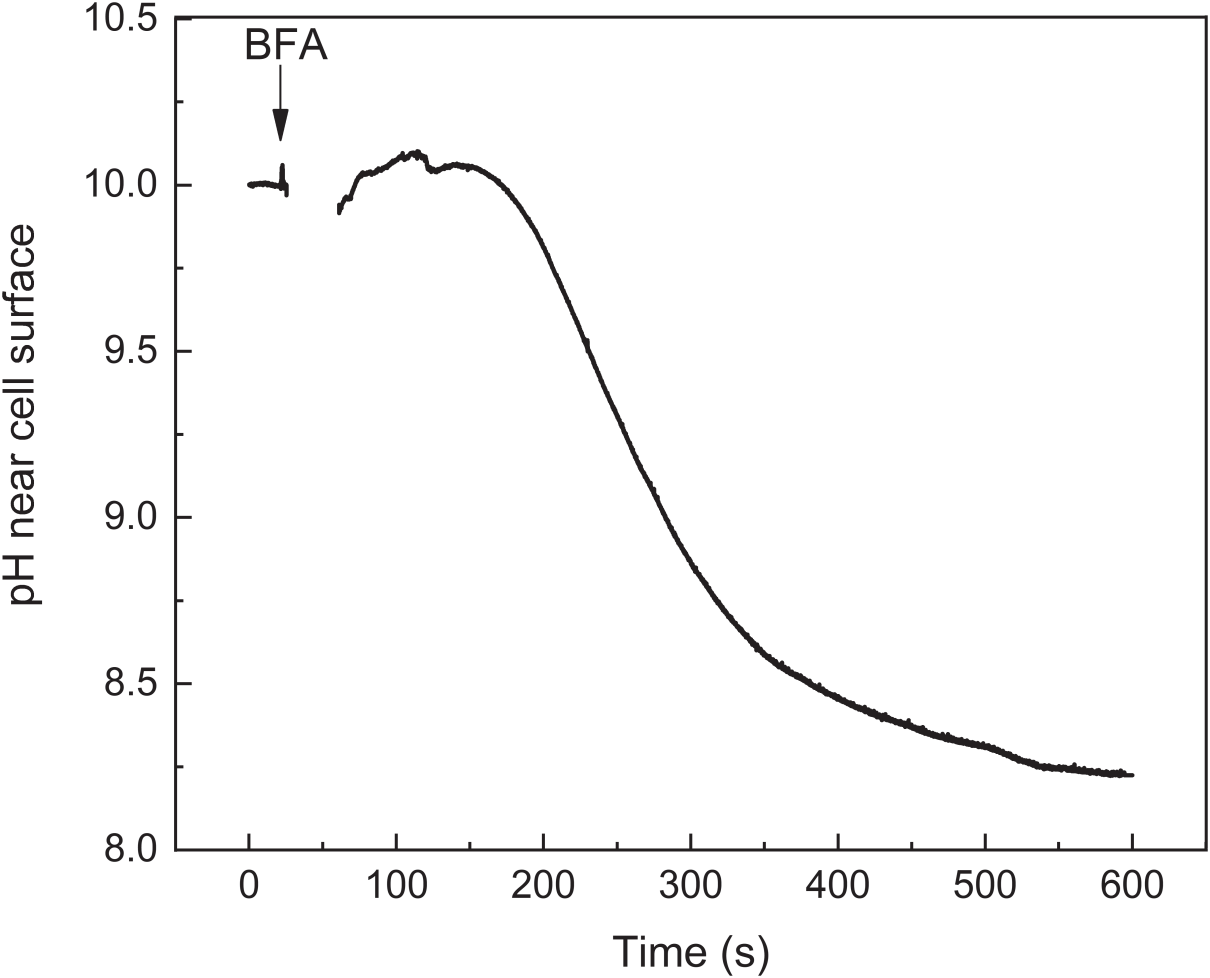
Inhibition of the alkaline band on the surface of illuminated *Chara* internodal cell (PFD 75 µmol m^−2^ s^−1^) after the replacement of standard medium (AFW) with AFW containing 0.1 mM BFA.

Fig. 4 shows that the replacement of standard AFW with AFW containing 0.1 mM BFA induced a large decrease in external pH in the alkaline zones even at a high PFD (75 µmol m^−2^ s^−1^). After replacing the medium, the outer pH (pH_o_) in the alkaline area first reestablished rapidly at the initial baseline (pH ∼ 10). After 2 min, pH_o_ started to decrease toward the weakly alkaline level (8–8.5) that was settled within 10 min. The streaming velocity and the effective PSII quantum yield were unaffected by the addition of BFA, which proves that the inhibition of a high pH zone resulted neither from the disturbance of cytoplasmic streaming nor from the suppression of photosynthetic electron flow. Thus, BFA appears as an effective although probably indirect inhibitor of H^+^ (OH^−^) transport in the alkaline zones.

**Figure 5:**
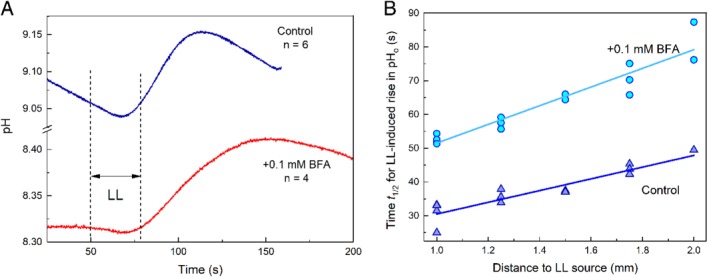
(A) Cyclosis‐mediated pH_o_ changes at the alkaline zone induced by local illumination in untreated *Chara* internodal cell (control) and after the replacement of the medium with AFW containing 0.1 mM BFA. The records on BFA‐treated cells were obtained after 20‐ to 40‐min incubation. Dashed vertical lines indicate the time of LL application (from 50 to 80 s). (B) The plots of a half‐time *t*_1/2_ of LL‐induced pH rise as a function of distance from the LL source in the absence of inhibitors (control) and after 20–120 min of incubation in the presence of 0.1 mM BFA.

After the treatment of internodes with 0.1 mM BFA, the ascending (rising) front of LL‐induced pH_o_ changes in the alkaline zones was strongly retarded (Fig. 5A). This retardation was manifested in the displacement of t_1/2_ and the position of the transient pH peak to longer times. The slope of the plot t_1/2_(d) became steeper, and the intercept with the y‐axis increased (Fig. 5B). Alteration of both parameters contributed to the delayed development of LL‐induced pH rise. In the acid zones of BFA‐treated cells after 2–3 h of incubation, we did not observe any decrease in pH upon the incidence of LL pulse.

### Effect of BFA on Chl fluorescence response to remote lighting

**Figure 6:**
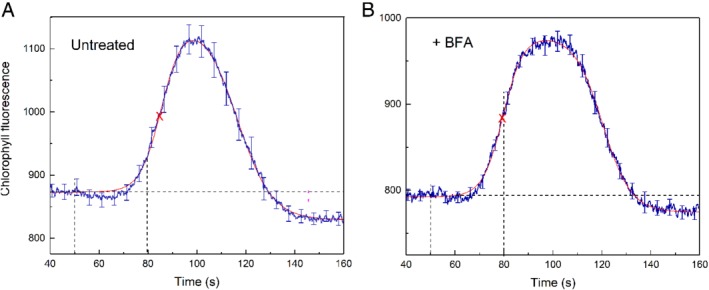
The influence of 0.2 mM BFA on cyclosis‐mediated changes of F′ Chl fluorescence induced by LL pulse applied at a 1.5‐mm distance upstream the analyzed cell region. Smooth red lines show the fit of experimental data with the sum of two Boltzmann functions. Vertical dashed lines delimit the period of LL pulse application. Crosses on the curves mark positions of t_1/2_ for the F′ increase. The F′ transient in (A) is an average trace (±se) obtained in a representative experiment with one cell (n = 6). (B) An averaged trace (±se) obtained for n = 5 on the same cell in the time frame from 35 to 70 min incubation with BFA is shown. The curve fit shown was superior compared to the approximation with the Gaussian curve.

The F′ fluorescence changes arising in response to LL were modified in a different way after the addition of BFA. They frequently became wider and acquired a trapezium‐like shape. The ascending front of F′ developed earlier in BFA‐treated than in untreated cells, and the F′ peak position shifted to shorter times. Both unaltered and trapezium‐shaped F′ responses were finely approximated with a sum of two Boltzmann functions. With an example of Fig. 6, the time t_1/2_ for the F′ rise, as counted from the onset of LL pulse, shifted from 35.6 s under control conditions to 29.6 s after adding 0.2 mM BFA. In this experiment, the velocity of cytoplasmic streaming was 86 µm s^−1^ and remained unaltered after the addition of BFA. Considering that the distance between the LL source and the area of measurements was 1500 µm, one may calculate that only the increase in streaming velocity up to 132 µm s^−1^ could potentially explain the advanced development (a 6‐s time shift) of the ascending F′ front. However, such streaming rates are unrealistic. Thus, the accelerated response of F′ cannot be attributed to the increased velocity of the fluid flow.

The fluorescence level achieved temporarily after the passage of F′ peak was below the initial baseline. Because of the decrease in F′ at this stage, the quantum yield of PSII‐driven linear electron flow calculated as (Fm′ − F′)/Fm′ was slightly higher (by 0.01 ± 0.004; mean ± sd) than before the application of LL pulse. This acceleration of linear electron flow concurrent with the decrease in the F′ baseline continued approximately for 3 min before the initial fluorescence and electron transport rates were established. It is possible that the delivery of reductants into the stroma of shaded chloroplast and the reductive modification of photosynthetic enzymes were involved in the temporary activation of linear electron flow.

**Figure 7:**
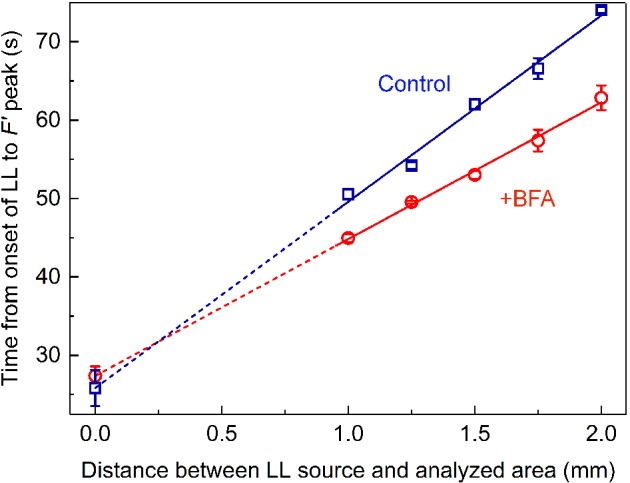
Time of F′ peak appearance (counted from the onset of LL) as a function of separation distance d between the centers of LL beam and the analyzed area in untreated cell and in the same cell incubated with 0.2 mM BFA for 0.5–3 h. Data are mean values and se determined in three to six replicate measurements.

The rates of lateral metabolite transmission involved in the distant control of Chl fluorescence were calculated from the plots of F′ peak position on a time scale against the distance between the LL source and the analyzed area (Fig. 7).

After the treatment of the cell with 0.2 mM BFA, the slope of the plot decreased significantly indicating the acceleration of signal transmission rate. At the same time, the value of graph intercept with the y‐axis (at d = 0) remained unchanged and equaled to 26–27.5 s. These results confirm that the rapid attainment of the F′ peak after the application of LL was entirely due to the increased rate of signal transmission with the fluid flow and was not caused by facilitation of other stages, such as the metabolite transport across the chloroplast envelope or its accelerated processing in the recipient chloroplasts.

**Figure 8:**
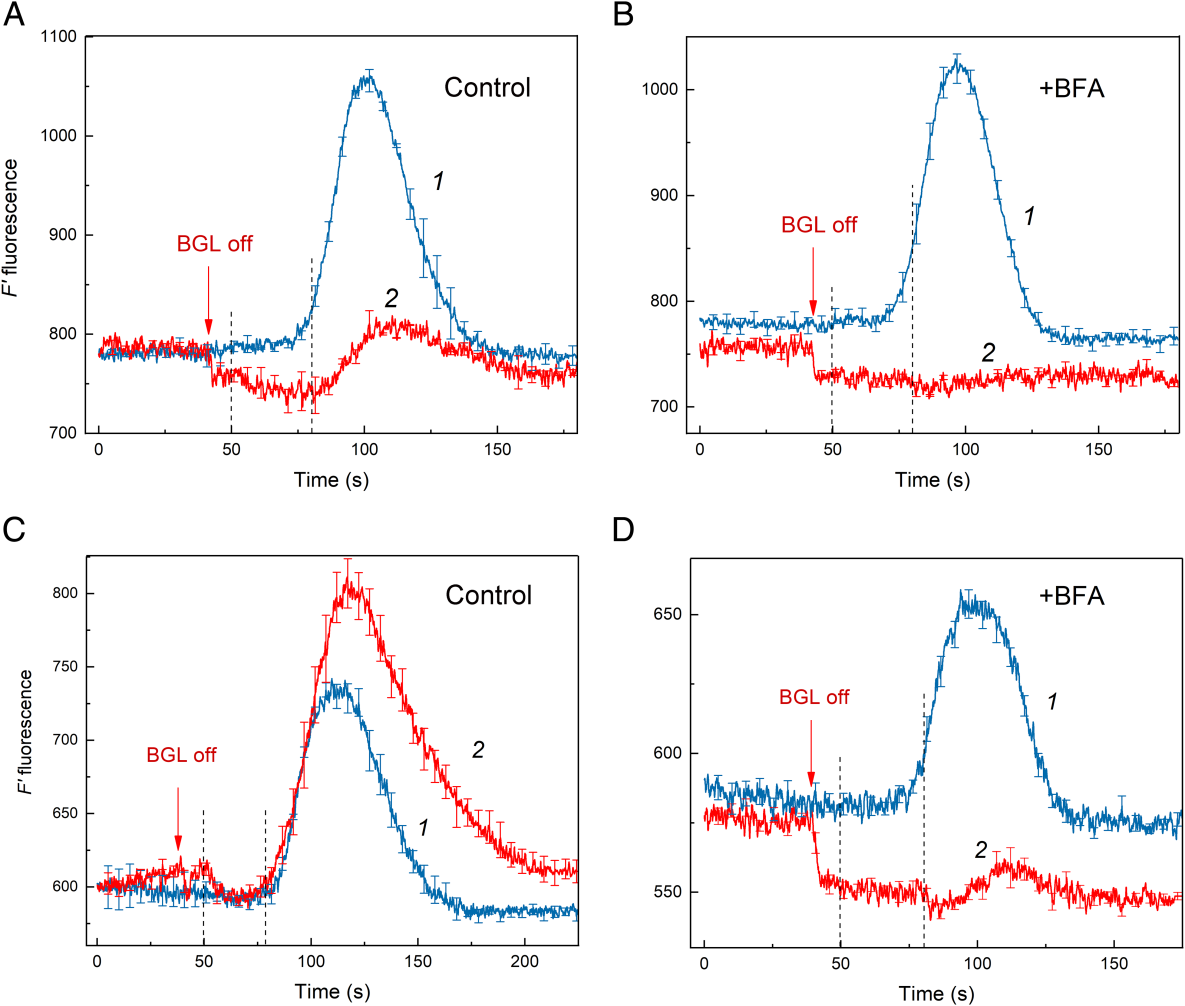
Cyclosis‐mediated changes in F′ Chl fluorescence induced by local illumination of a remote cell region under background illumination of the whole cell (curves 1) and after darkening (curves 2). The graphs (A,B) and (C,D) were obtained with internodal cells showing different amplitudes of LL‐induced fluorescence changes after darkening. (A,C) Control conditions; (B, D) after 2–3.5 h of incubation in the presence of 0.2 mM BFA. Arrows refer to traces 2 only and indicate the moment when BGL was switched off shortly before the onset of a 30‐s pulse of LL. Average kinetics (±se) are presented with n = 4–7. Dashed vertical lines indicate the time of LL application (from 50 to 80 s).

Although BFA accelerated the microfluidic communications between spatially remote chloroplasts under weak background illumination, the interplastid communications in the absence of BGL were strongly suppressed. Fig. 8 shows the action of BFA on F′ fluorescence transients induced by LL pulse under dim BGL (curves 1) and shortly after switching off BGL (curves 2) in untreated internodal cell (Fig. 8A) and in the same cell incubated for 2–3.5 h in the presence of 0.2 mM BFA (Fig. 8B). Routinely, the amplitude of LL‐induced F′ changes in darkened chloroplasts is approximately threefold lower than in chloroplasts illuminated with dim BGL (Fig. 8A). The F′ response of darkened chloroplasts also features a relatively slow decline of fluorescence after the peak. The deceleration of F′ decline is conceivable because the oxidation of plastoquinone (PQ) pool underlying this process relies largely on the activity of PSI that does not operate in darkness. A slow oxidation of PQ and Q_A_ (primary quinone acceptor in photosystem II) in darkness is mediated by the plastid terminal oxidase (Krieger‐Liszkay and Feilke 2016, Shikanai 2016).

The treatment of cell with BFA had little effect on the LL‐induced F′ response under background illumination but strongly suppressed the F′ changes in a darkened cell (Fig. 8B). Fig. 8C shows a rare case when the response of F′ to LL in the absence of BGL was larger than under BGL. The large amplitude of this F′ change suggests that non‐photochemical mechanism of PQ reduction contributes significantly to the reduction of PQ by reducing substances imported from the cytoplasmic flow. Large variations in the amplitude of LL‐induced F′ changes in darkened plastids are not yet understood. Nevertheless, even in the case shown in Fig. 8C, the F′ response to LL in BFA‐treated cell decreased much stronger in the absence of BGL than under dim BGL (Fig. 8D).

### Effect of BFA on endosome trafficking and distribution in *Chara* internodal cells

The effect of BFA on the abundance and morphology of organelles in *Chara* cytoplasm was studied with FM 1‐43, a non‐permeant fluorescent styryl dye, which incorporates in the plasma membrane from where it is taken up via endocytic vesicles (Fig. S2). The endocytic vesicles fuse with and deliver the dye to the TGN. From the TGN, the FM 1‐43‐stained membrane is further passed on to multivesicular bodies (MVBs) or to recycling endosomes. MVBs are destined for degradation and fuse with the vacuolar membrane. Recycling endosomes travel back to and eventually fuse with the plasma membrane, especially during wound healing (Klima and Foissner 2011). This allows studying membrane trafficking of selected organelles also in characean internodes that are so far refractory to genetic manipulation. To allow an undisturbed view on the endoplasm, where most of the FM‐stained organelles are located, chloroplasts were removed locally by irradiation with intense light 1 day prior to the experiments (see section Material and methods).

**Figure 9:**
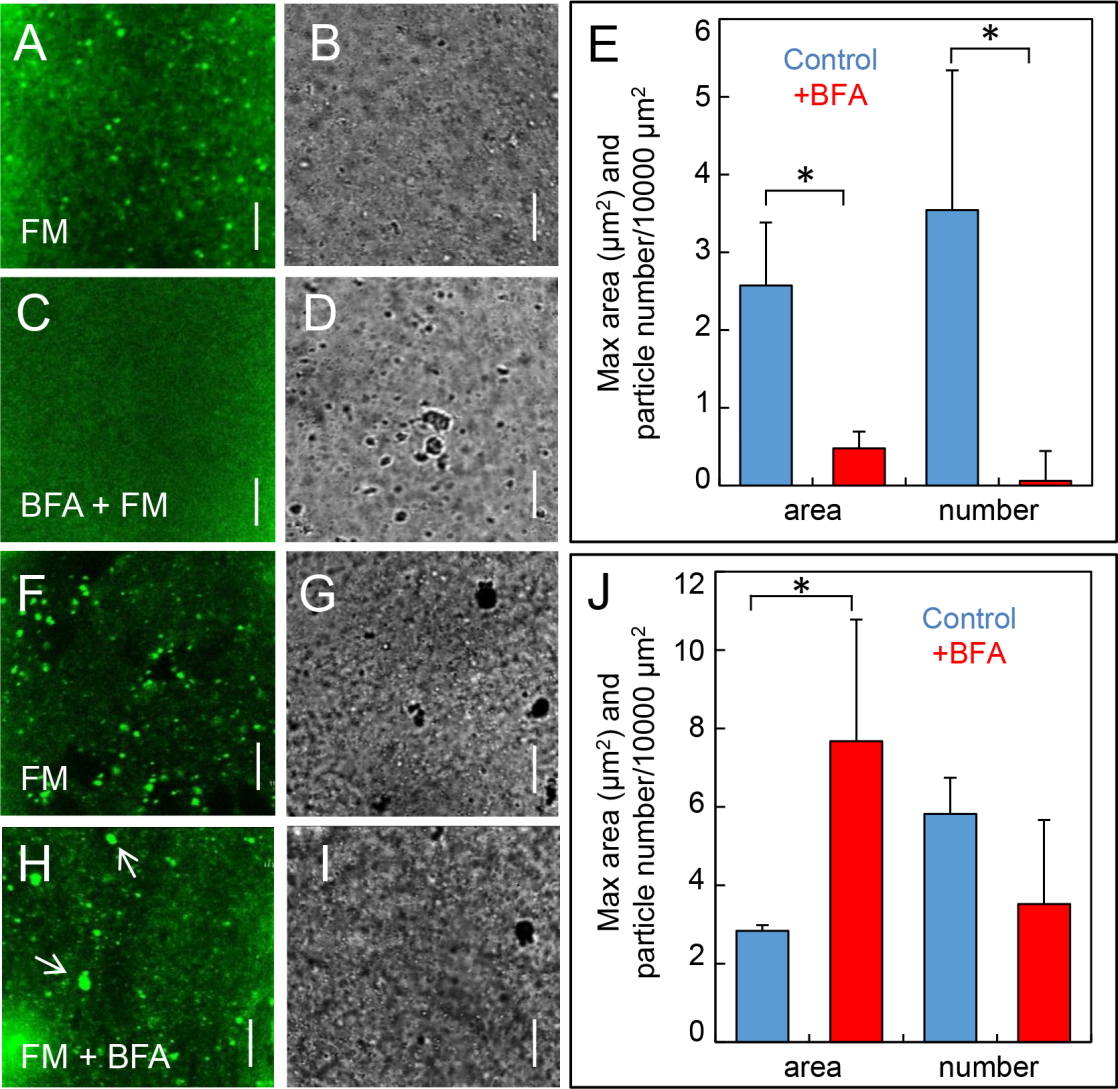
Effect of BFA on FM 1‐43‐stained organelles in the endoplasm of *Chara* internodal cells. (A–E) Effect of BFA on FM internalization. Cells were pulse‐labeled for 2 min with FM 1‐43 dissolved in 0.2 mM BFA or in control solution. Images were taken 10–20 min after further incubation in dye‐free medium. In control cells, numerous FM 1‐43‐stained organelles are present in the streaming endoplasm (A fluorescent image, B bright field image). In BFA‐treated cells, no fluorescent particles are visible (C,D). BFA significantly inhibits the uptake of FM 1‐43 via endosomes (number of particles per 10 000 µm^2^ endoplasm) and decreases the maximum particle size per video image (E). (F–J) Effect of BFA on existing organelles. Cells were pulse‐labeled with FM 1‐43 for 4 min and left in AFW to allow staining of endosomes. After 15 min, cells were treated with control solution (F,G) or with 0.2 mM BFA for 1 h (H,I; arrows point to enlarged particles). The significant BFA‐induced increase in maximum particle size per video image reflects the formation of BFA compartments (J). Data are means ± sd from at least three cells (videos with 50–60 images each); asterisks indicate significance at *P* ≤ 0.05 (two‐tailed *t*‐test). A, C, F and H are the fluorescent images, B, D, G and I are the corresponding bright field images. Bars are 10 µm.

At first, we pulse‐labeled control cells for 2 min and followed the appearance of fluorescent organelles. After 10–20 min, numerous FM 1‐43‐stained organelles were carried along with the streaming endoplasm (Fig. 9A,B). Their mean maximum size per video image was between 2 and 3 µm^2^ (Fig. 9E) suggesting that the internalized plasma membrane dye had already reached the TGN and the MVBs which have comparable dimensions. However, when cells were pulse‐labeled with FM 1‐43 dissolved in 0.2 mM BFA and incubated in the BFA solution for 10–20 min, the endoplasm appeared empty, apart from very few and tiny fluorescent particles (Fig. 9C–E). Hence, BFA nearly completely arrested endocytosis in *Chara* internodal cells and, because of the lack of FM 1‐43 internalization it was not possible to visualize the TGN which is a major component of the BFA compartments.

Next, we investigated the effect of BFA in cells, which were stained before inhibitor treatment. Images were obtained after an incubation time of about 60 min in order to allow the gathering of organelles and the formation of BFA compartments. Control cells contained abundant fluorescent organelles with a maximum mean area (size) similar to that observed after 10–20 min (Fig. 9F,G and J compared with A,B,E). BFA‐treated internodes contained significantly larger fluorescent organelles (BFA compartments) and the relative number of all FM 1‐43‐stained organelles was lower than in untreated cells, although not significantly (Fig. 9H–J).

### Effect of BFA on intercellular transmission of cyclosis‐distributed photometabolites

**Figure 10:**
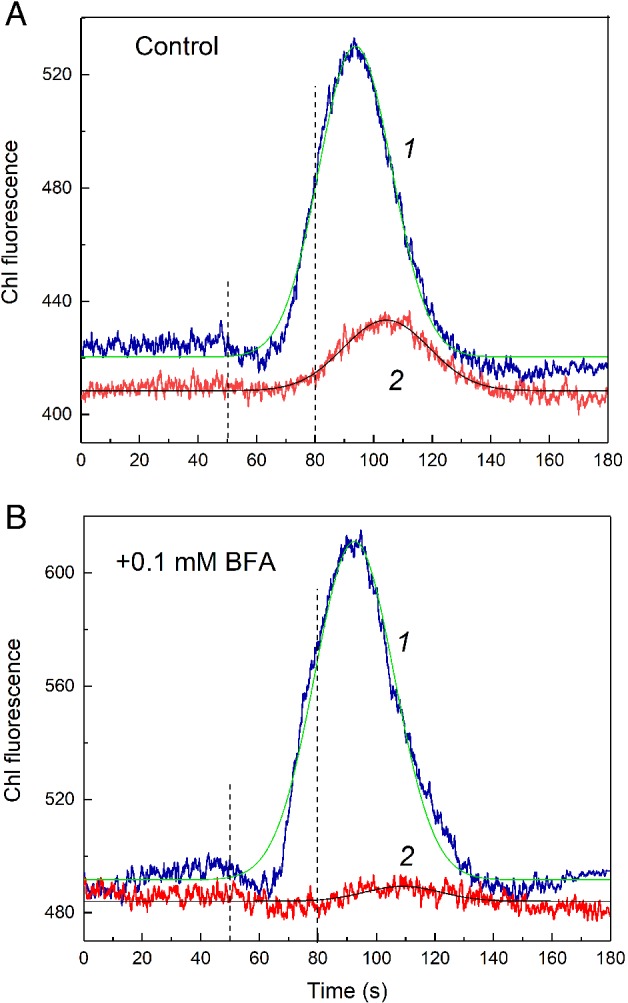
Effect of 0.1 mM BFA on intercellular transmission of cyclosis‐distributed photometabolites. Chl fluorescence changes were induced by local illumination of a remote cell region located within the same cell (curves 1) and in the neighbor internodal cell (curves 2). The area under the F′ curve was taken as a measure for the amount of regulatory metabolite delivered to chloroplasts of analyzed area. Smooth thin lines are approximations of data with Gaussian curves. Curves are averaged data and se for n = 8. Dashed vertical lines indicate the time of LL application (from 50 to 80 s).

The intercellular vesicle trafficking was reported to deliver some protein constituents to the plasmodesmata in a BFA‐sensitive manner (Thomas et al. 2008). We tested whether BFA affects the intercellular transmission of the photometabolites carried by the streaming cytoplasm (Bulychev 2019). The intercellular transmission was judged from the comparison of Chl fluorescence (F′) transients observed in shaded cell areas after the application of LL pulse on the upstream cell region in two measuring configurations. In one configuration, the locally illuminated and analyzed regions were located within the same cell and separated by a distance of 1 or 1.5 mm. In the other configuration, the illuminated and analyzed regions were separated by the same distance but located in different internodes. Fig. 10 shows the LL‐induced F′ transients for the intercellular (*cis*) and transcelluar (*trans*) configurations in the absence of inhibitors (control conditions) and within 1 h of incubation in the presence of 0.1 mM BFA.

In physiological (control) conditions, the amount of photometabolites delivered to chloroplasts in *cis* configuration (Fig. 10A, area under the curve 1) was approximately 3‐ to 3.7‐fold higher than in the *trans* configuration (Fig. 10A, area under the curve 2). The shifted (∼10 s) F′ peak positions on curves 1 and 2 indicate the time of metabolite passage through plasmodesmata in the nodal complex. The incubation of cells with 0.1 mM BFA inhibited the transcellular delivery of cyclosis‐distributed photometabolites to chloroplasts of the sink cell and, at the same time, slightly promoted their delivery to chloroplasts of the source cell. For example in the experiment shown in Fig. 10B, the area under the curves 1 increased after BFA addition by 18.5%, whereas the area under the curves 2 reduced more than fivefold. A milder suppression of the cell‐to‐cell passage of cytoplasmic metabolites (by a factor of 1.4–2.0) was observed in three out of seven experiments; the transcellular permeation of photometabolites in one cell turned out insensitive to BFA. The selective inhibition of transcellular movement of photoassimilates and/or reducing equivalents in the majority of tested cells indicates the involvement of vesicular trafficking in the maintenance of functional plasmodesmata in characean algae. The inhibition of transcellular permeation of photometabolites was partly reversible upon washing the cell with fresh AFW. Up to 80% recovery in the area under the F′ curve measured in the *trans* configuration was noted (not shown).

## Discussion

Under control conditions in the absence of BFA, local illumination of *Chara* internodal cells induces changes of Chl fluorescence and pH_o_ in remote cell regions that proceed in identical time ranges (Fig. 3A). The rates of lateral transmission of the metabolites regulating the plasmalemmal H^+^ transport and the chloroplast activities (assesses from slopes of straight lines in Figs 2 and 3B) were similar after 20–40 min treatment with BFA. Hence, the activation of plasma membrane ‘high pH channels’ and H^+^‐pump, as well as the reduction of photosynthetic electron transport carriers upon local illumination of a remote cell region are caused by the photosynthetic metabolites transported in the cytoplasmic flow at equal velocities. However, the activation of H^+^ efflux in ATPase‐enriched regions occurred in advance to the activation of inward H^+^ flux (or OH^−^ efflux). The time‐shifted promotion of H^+^‐pump activity and passively conducting channels might reflect the existence of two metabolites released by illuminated chloroplast after different lag periods. Accordingly, there will be a delay in the interactions of these metabolites with the PM transporters.

The incubation of internodal cells in the presence of BFA was found to accelerate the delivery of reducing substances from brightly illuminated chloroplasts into the stroma of shaded plastids with the concomitant deceleration of LL‐induced recovery of alkaline bands. One explanation of such reciprocal influence of BFA on chloroplast and PM activities is that the enhanced delivery of the photometabolites into the chloroplasts attenuates the supply of the shared metabolite to the PM transporters. The reciprocal influence also excludes the possibility that variations in the rate of cytoplasmic streaming are responsible for this effect (Bulychev et al. 2018). Long‐range interaction of chloroplasts was not restricted to single cell level (Bulychev 2019). Photometabolites were also transmitted to neighbor internodal cells in a BFA‐sensitive manner.

**Figure 11:**
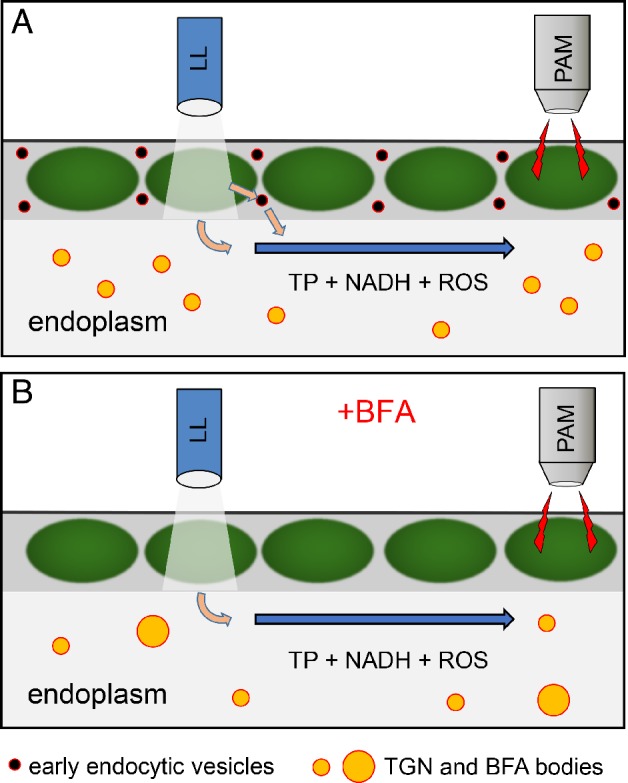
Effects of BFA on long‐distance interaction between chloroplasts in *Chara* internodal cells. (A) Under control conditions, local illumination (LL) causes the release of photometabolites which bind to the membranes of early endocytic vesicles in the cortex and to the membranes of the TGN and other organelles in the streaming endoplasm. The intermediary steps of reversible binding to vesicles in the stationary ectoplasm prolong the total time of metabolite delivery with the streaming endoplasm. Non‐absorbed and desorbed metabolites are carried along with the streaming endoplasm (arrow) and induce changes of Chl fluorescence measured by PAM in remote shaded cell regions. (B) BFA inhibits the formation of early endocytic vesicles and causes the agglomeration of TGN and other endoplasmic organelles thereby reducing the surface area available for binding of photometabolites. The elimination of binding–unbinding to cytoplasmic vesicles accelerates the response of remote chloroplasts. Rate of cyclosis, represented by the length of arrows, is not affected by BFA.

In most animal and plant cells, including those of *Arabidopsis*, BFA inhibits the delivery of vesicles toward the cell periphery and their fusion with the plasma membrane (exocytosis; Robinson et al. 2008). In internodes of *Chara*, exocytosis is not affected (own unpublished data) but endocytosis is arrested and the relative number of early endocytic vesicles is significantly lower than in controls (Fig. 9A–E). In addition, BFA induces the formation of BFA compartments. The BFA compartments are aggregates and fusion products of various organelles (Golgi, TGN and ER) and their remnants (see section Introduction). In our experiments, the formation of BFA compartments came along with a slight reduction in the relative number of FM‐stained particles. It must be noted, however, that the size and number of the BFA compartments visualized with styryl dyes is likely to be underestimated because the fluorescent dye labels only the TGN but not the Golgi or the ER cisternae (Fig. S2). In any case, the BFA‐induced arrest of endocytosis, i.e. the absence of early endocytic vesicles, and the agglomeration and fusion of organelles will considerably reduce the membrane surface available for binding with photometabolites (Fig. 11). In previous studies, we applied wortmannin, an inhibitor of PIP3 and PIP4 kinases which induces the formation of large aggregates consisting of TGN and MVBs (Foissner et al. 2016). Wortmannin reduced the amplitude of pH peaks (Bulychev and Foissner 2017) but, in contrast to BFA, had only minor effects on long‐distance transmission of photometabolites (Bulychev and Foissner 2017, own unpublished data). This may indicate that chloroplast‐released substances bind (preferentially) to the membrane of specific organelles.

Unlike the inhibition of LL‐induced Chl fluorescence changes in the absence of BGL, which develop on a longer time scale, the suppression of pH bands and the retardation of LL‐induced pH_o_ changes can be regarded as fast responses. The LL‐induced pH rise is decelerated already within few minutes after BFA addition and cannot be explained by the formation of BFA bodies, which is a longer lasting process extending over at least 30 min in *Chara*. The fast response can, however, be correlated with the immediate arrest of endocytosis. It is conceivable that transporters with short lifetimes must be continuously recycled which is not possible in the presence of BFA. For the plasma membrane H^+^ ATPase, an apparent half‐life of around 12 min has been reported (Hager et al. 1991), which approximately corresponds with the time required to significantly reduce the pH at an alkaline band in this study (Fig. 4). Consistently, wortmannin also inhibits both pH banding and endocytosis in *Chara*, although to a lesser extend (Foissner et al. 2016, Bulychev and Foissner 2017). An argument against the hypothesis that continuous renewal of transporters is required to maintain ion transport is, that ikarugamycin, another inhibitor of endocytosis, has no effect on pH banding in *Chara* (own unpublished data). However, ikarugamycin is far less effective than BFA and never completely arrests constitutive endocytosis, even at the highest concentration tested (Hoepflinger et al. 2017). A more direct influence of BFA on membrane transporters or membrane properties, independent of vesicle fusion and fission effects, must also be considered. BFA was reported to have channel‐like properties when inserted in artificial membranes (Zizi et al. 1991) but BFA does not affect the membrane potential in *Chara* internodal cells up to a concentration of 25 µM (own unpublished data) which argues against this possibility. Finally yet importantly, BFA may influence chloroplast activity and metabolism via inhibition of vesicle formation destined for fusion with chloroplasts (Kitajima et al. 2009). It has been demonstrated that BFA prevents the delivery of nucleotide pyrophosphatase/phosphodiesterases to the chloroplasts (Kaneko et al. 2016) and that there is a marked increase in starch accumulation in plastids from different cell types after Golgi disassembly promoted by BFA or through an inducible Sar1‐GTP system (Hummel et al. 2010). Clearly, further investigation is required to clarify how BFA affects the activity of chloroplasts and transporters in *Chara* and other cells.

## Author contributions

Both authors contributed equally to the design of the experiments and writing of the manuscript, and conducted the experiments together.

## Supporting information


**Fig. S1.** Schematic view of a *Chara* internodal cell.

**Fig. S2.** Major vesicle trafficking pathways in a plant cell.

## Data Availability

The authors declare that all data supporting the findings of this study are available in the paper.
