# Supplementary material for: Inhibition of endosomal trafficking by brefeldin A interferes with long‐distance interaction between chloroplasts and plasma membrane transporters
Source: Physiol Plant. 2019 Dec 26;169(1):122–34. doi: 10.1111/ppl.13058 (PMC7216902; doi:10.1111/ppl.13058)
Supplement: Supplementary file 1 — Fig. S1. Schematic view of a Chara internodal cell. [file PPL-169-122-s001.pdf]

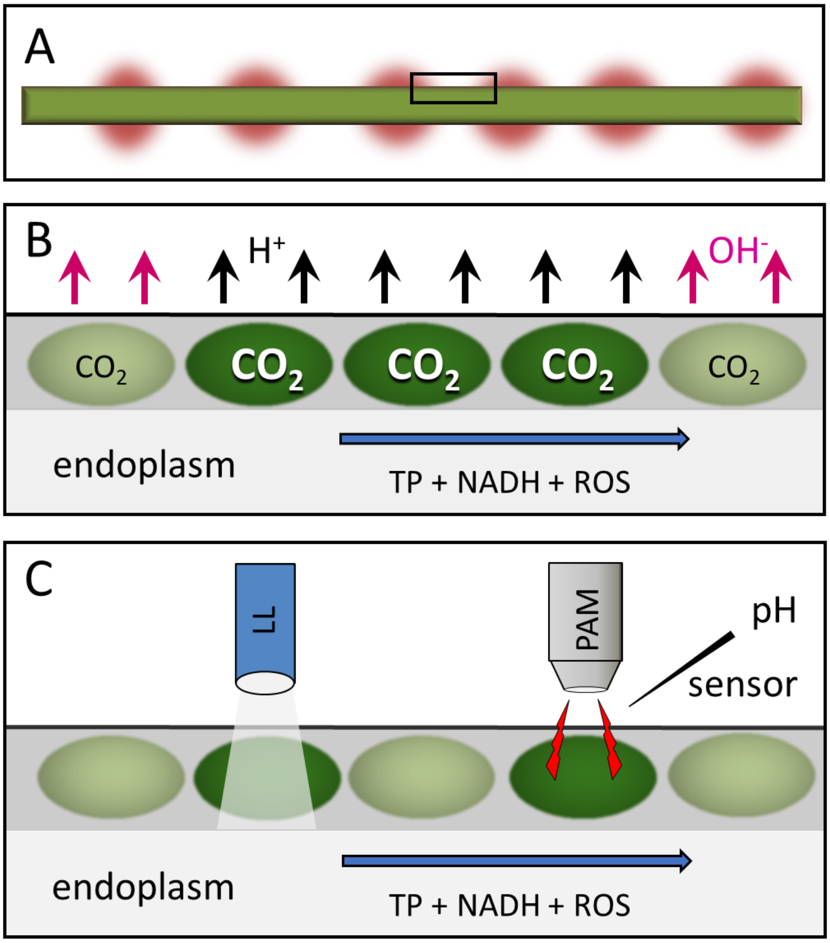

Fig.S1. Schematic view of a *Chara* internodal cell showing pH banding, cellular organization, ion and metabolite flows and experimental setup. (A) The pH banding pattern of internodal cells can be visualized by pH indicating phenol red; pink regions indicate alkaline pH. (B) Detail of internodal cell according to inset in A. (B) The longitudinal section (not drawn to scale and without cell wall) shows the outer layer with stationary chloroplast files and the circulating endoplasm (horizontal arrow). When exposed to overall illumination plasma membrane bound H^+ ATPases release protons into the medium, which increases the availability of CO_2 and chloroplast activity at the acid regions. Current evidence suggests that photometabolites and reactive species (ROS) produced during photosynthesis are transported within the streaming endoplasm and activate “high pH channels” to ensure overall pH homeostasis of the cytoplasm. (C) Local illumination (LL) causes local release of triosephosphates (TP) and NADH, which interact with the photosynthetic electron transport chain of downstream-located chloroplasts. Their activity (chlorophyll fluorescence, red lightnings) can be measured with PAM microscopy. Changes in external pH are detected with pH electrodes (compare Bulychev and Komarova 2017).
