# Supplementary material for: Inhibition of endosomal trafficking by brefeldin A interferes with long‐distance interaction between chloroplasts and plasma membrane transporters
Source: Physiol Plant. 2019 Dec 26;169(1):122–34. doi: 10.1111/ppl.13058 (PMC7216902; doi:10.1111/ppl.13058)
Supplement: Supplementary file 2 — Fig. S2. Major vesicle trafficking pathways in a plant cell. [file PPL-169-122-s002.pdf]

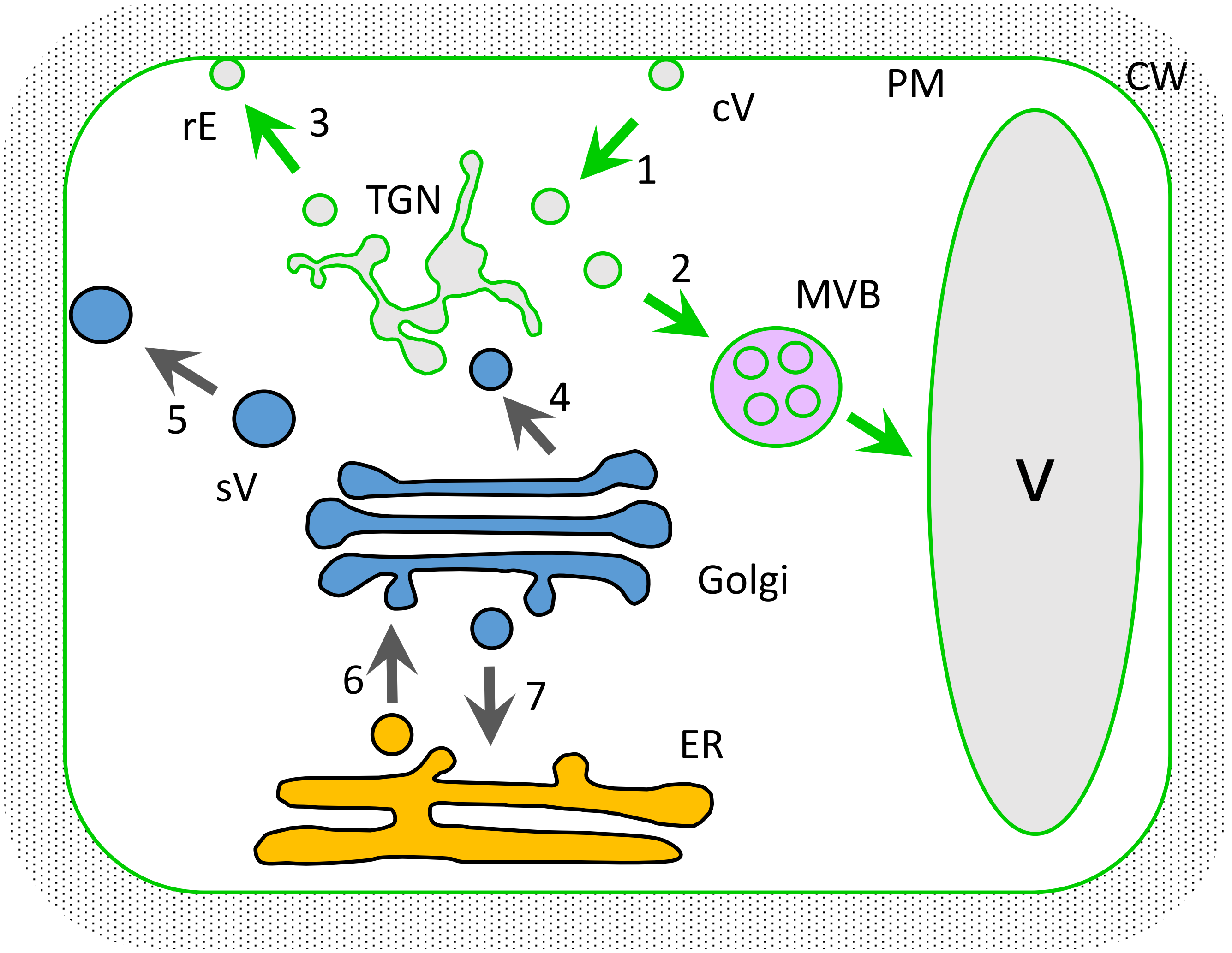

Suppl. Fig. 2. Major vesicle trafficking pathways in a plant cell. 1-3 Pathways stained by FM dyes (green), 4-7 pathways not stained by FM dyes. 1 The FM dye incorporates into the plasma membrane (PM) and is internalized via coated vesicles (endocytosis) which fuse with the trans-Golgi network (TGN). 2 The TGN gives rise to the multivesicular bodies which pass on the dye to the membrane of the vacuole (V). 3 Recycling endosomes (rE) from the TGN may fuse with the PM (exocytosis). 4 The Golgi delivers the material for the TGN. 5 Golgi-derived secretory vesicles fuse with the PM (exocytosis). 6 The Golgi itself forms by fusion of vesicles released from the endoplasmic reticulum (ER). 7 Golgi membranes and cargo are in part rescued back to the ER (retrograde trafficking). CW = cell wall
